# Mitochondrial bioenergetics and intracellular calcium concentration in primary myotubes from mouse models of malignant hyperthermia

**DOI:** 10.1016/j.bja.2025.05.060

**Published:** 2025-08-19

**Authors:** Vikas Kaura, Leon Chang, Philip Morgan Hopkins, Marie-Anne Shaw

**Affiliations:** 1 Leeds Institute of Medical Research at St James’s, University of Leeds, Leeds, UK; 2 Department of Anaesthesia, Leeds Teaching Hospitals NHS Trust, Leeds, UK; 3 Malignant Hyperthermia Unit, St James’s University Hospital, Leeds, UK

**Keywords:** bioenergetics, malignant hyperthermia, mitochondria, myotubes, ryanodine receptor, *RYR1*

## Abstract

**Background:**

Malignant hyperthermia (MH) is a potentially fatal hypermetabolic reaction to general anaesthesia arising from skeletal muscle calcium dysregulation. Previous studies of resting cells support an association between MH susceptibility, mitochondrial dysfunction, and defects in fatty acid metabolism, which are understood to be downstream consequences of calcium dysregulation. We hypothesised that in mouse models of MH susceptibility, genotypes associated with higher cytoplasmic calcium concentrations would have a proportionally higher mitochondrial oxygen consumption rate (OCR). We aimed to test this and validate a cell-based assay system.

**Methods:**

A high-throughput mitochondrial assay was used to compare OCR between myotubes derived from control and three different genotypes of mice containing ryanodine receptor 1 variants (p.G2435R heterozygous and homozygous, p.T4826I heterozygous) that confer susceptibility to MH.

**Results:**

Baseline comparisons showed effects of genotype on OCR (*P*<0.0001), with *Ryr1* p.G2435R homozygous myotubes having the highest basal normalised OCR (*P*<0.01). *Ryr1* p.G2435R homozygous required a greater proportion of basal respiration to produce adenosine triphosphate (ATP), and had a higher proton leak and greater non-mitochondrial OCR (*P*<0.01). All genotypes except *Ryr1* p.G2435R homozygous were primarily dependent on the glucose/pyruvate pathway for achieving their maximal OCR upon uncoupling.

**Conclusions:**

The high-throughput method used produced data consistent with findings in skeletal muscle fibres, but with a greater sensitivity to genotypic effects. This validates the use of cultured myotubes in lieu of muscle fibres in studying mitochondrial bioenergetics in models of MH, and indicates that mitochondrial bioenergetics are not directly affected by myoplasmic calcium concentrations in young MH mice.

**Editor’s key points**

• A sensitive high-throughput mitochondrial assay was used to compare oxygen consumption rate between myotubes from control mice and mutants containing ryanodine receptor 1 variants of human malignant hyperthermia.
• Data from myotubes confirmed genotype-dependent effects on basal metabolic rate and proton leakage, validating the Seahorse analyser as an alternative high-throughput method for studying mitochondrial bioenergetics in malignant hyperthermia.
• The degree of change in mitochondrial bioenergetics was partly influenced by intracellular [Ca^2+^] in myotubes, with basal mitochondrial activity influenced in a gene-dose-dependent manner. However, the mitchondrial bioenergetics in p.T4826I heterozygous cells were similar to wild-type cells.
• Future high-throughput cell-based studies using both animal models and human malignant hyperthermia cells might help clarify relationships between mitochondrial bioenergetics and human phenotypes.

Malignant hyperthermia (MH) is a skeletal muscle pharmacogenetic condition in which underlying calcium dysregulation results in a hypermetabolic response after exposure to inhalation anaesthetics and suxamethonium.^1^^,^^2^ Such reactions can be fatal, but in the absence of triggering agents, MH-susceptible individuals typically do not show an overt clinical phenotype.^3^ However, some MH-susceptible patients report myopathic traits such as muscle weakness and exercise intolerance, which suggests underlying metabolic dysfunction;^4^ this has led to research investigating basal skeletal muscle metabolism in MH-susceptible muscle.

Studies on humans have been limited, but evidence thus far supports an association between MH-susceptible status, mitochondrial dysfunction, and defects in fatty acid metabolism.^5^, ^6^, ^7^, ^8^ Lavorato and colleagues^6^ first observed more structural deformities and swelling in mitochondria of MH-susceptible skeletal muscle by electron microscopy, thus implying a degree of functional defect. Chang and colleagues^7^ used high-resolution respirometry (Oroboros analyser) to investigate activity of the electron transport chain and to show evidence of mitochondrial dysfunction in MH-susceptible muscle through measurements of oxygen consumption rates (OCRs). Bojko and colleagues^8^ conducted metabolomic profiling on human MH-susceptible muscle to show accumulation of various lipid metabolites consistent with a defect in lipid metabolism and a shift in metabolite usage. These defects in metabolism are understood to be downstream consequences of the Ca^2+^ dysregulation, which is a hallmark of MH susceptibility.^3^

Intracellular Ca^2+^ concentration ([Ca^2+^]) in MH-susceptible skeletal muscle is dysregulated owing to pathogenic variants in *RYR1,* the gene encoding the sarcoreticular calcium release channel ryanodine receptor 1 (RyR1), which accounts for >70% of MH-susceptible cases.^9^ Pathogenic variants in *RYR1* result in Ca^2+^ leak from the sarcoplasmic reticulum (SR), which is associated with up to a three-fold elevation in intracellular [Ca^2+^] relative to non-MH-susceptible (MHN) muscle.^10^^,^^11^ To mimic this cellular environment and explore the effects of specific mutations, numerous MH-susceptible *Ryr1* knock-in mouse models have been developed.^2^^,^^12^, ^13^, ^14^ MH mouse models carrying *Ryr1* p.R163C and p.T4826I are associated with a more severe MH phenotype and have elevated intracellular [Ca^2+^] of ∼300 nM in skeletal muscle from heterozygous animals compared with ∼120 nM in wild-type (WT) control mice. In contrast, the *Ryr1* p.G2435R model, based on the most common MH causative variant in the UK, has a milder phenotype with a mean intracellular [Ca^2+^] of 156 nM in heterozygous and 235 nM in homozygous animals.^2^

Studies on each MH mouse model have consistently reported evidence of mitochondrial dysfunction and increased oxidative stress.^15^, ^16^, ^17^, ^18^, ^19^, ^20^ On the basis of these findings, we hypothesised that leaky RyR1 channels cause elevated myoplasmic [Ca^2+^] mediated by increased extracellular Ca^2+^ entry.^21^ Chronic elevations in myoplasmic [Ca^2+^] lead to a cascade involving increased reactive oxygen species (ROS) and reactive nitrogen species (RNS) production, which through binding to RyR1 reduce its sensitivity to Ca^2+^. The loss of Ca^2+^ inhibition on RyR1 leads to greater ROS and RNS together with elevated mitochondrial Ca^2+^[Ca^2+^] with an ensuing increase in mitochondrial ATP synthesis and basal metabolic stress.^16^, ^17^, ^18^, ^19^, ^20^, ^21^, ^22^ One study that contradicts this hypothesis, found that despite the higher intracellular [Ca^2+^] of 300 nM in skeletal muscle fibres from *Ryr1* p.T4826I heterozygous animals, they had similar mitochondrial activity to WT animals.^20^

In this study, we adopted a high-throughput method using the Agilent Seahorse XFe96 (Agilent Technologies, Santa Clara, CA, USA) to investigate mitochondrial bioenergetics in skeletal muscle myotubes, and to assess the consistency of outcomes with those from the Oroboros analyser (Oroboros Instruments, Innsbruck, Austria).^23^^,^^24^ Comparable results would further validate the use of myotubes to model the bioenergetics in skeletal muscle fibres from different mouse models of MH. Such a system would provide several advantages including high throughput, the ability to study different oxidative pathways that are difficult to probe on the Oroboros platform, reduced cost, and reduced use of animal experimentation.

## Methods

### Myoblast isolation and maintenance

After ethical approval, myoblasts were isolated from knock-in mice containing the *Ryr1* variants p.G2435R/WT (Het), p.G2435R/p.G2435R (Hom), p.T4826I/WT (T4826I Het), and WT controls as described.^2^ Briefly, skeletal muscle was isolated from forelimbs and hindlimbs of knock-in and control mice, was enzymatically digested, underwent red cell lysis, was filtered through cell strainers, and was washed and centrifuged at 300 x *g*. The ensuing pellet was resuspended in Ham’s F10 media with GlutaMAX, 20% fetal bovine serum (FBS), 400 units ml^−1^ with penicillin and streptomycin (all from Gibco, ThermoFisher Scientific, Waltham, MA, USA), then plated onto 10 cm Petri dishes (Cell+, Sarstedt, Nümbrecht, Germany), and incubated at 37°C in 95% air/5% CO_2_. Skeletal muscle myoblasts were preferentially selected by pre-plating at least six times before being placed onto entactin-collagen IV-laminin (ECL) cell attachment matrix (Merck KGaA, Darmstadt, Germany) coated Petri dishes, and fibroblast growth factor-β (FGF-β; 2.5 ng ml^−1^) added.^25^ Pure myoblasts were maintained on rat tail collagen (2 μg cm^−2^) coated Petri dishes in mouse proliferation medium consisting of Ham’s F10 with 20% FBS, penicillin and streptomycin (200 units ml^−1^), and FGF-β (2.5 ng ml^−1^), with media changes every 1–2 days.

### Myoblast plating and differentiation in Seahorse plates

Myoblasts were plated at 40×10^3^ cells (automated cell counter quantification; TC10, BioRad, Hercules, CA, USA) per well in proliferation media on XFe96 cell culture microplates (Agilent Technologies) pre-coated with growth factor reduced extracellular matrix from Engelbreth-Holm-Swarm murine sarcoma (21 mg per well, Merck KGaA, Darmstadt, Germany). Myoblasts were briefly centrifuged onto the plate for 30 s at 300 x *g* and placed in a humidified incubator at 37°C with 5% CO_2_ 95% O_2_ mix. After 12 h the media was substituted to differentiation media consisting of Dulbecco's modified essential media (DMEM) with 4 mM GlutaMAX, 4.5 g L^−1^ glucose, 1 mM pyruvate, 2% heat inactivated horse serum (Gibco, ThermoFisher Scientific), and 100 U units ml^−1^ with penicillin and streptomycin. Media changes occurred every 1–2 days, with myotubes assayed on day 3 of differentiation.

All genotypes produced multinucleated myotubes after 3 days of differentiation, and these wells were included in the analysis. Previous studies in our laboratory using myoblasts from these genotypes^2^ have confirmed that the multinucleated cells express myosin heavy chain and have functioning excitation-calcium release coupling (Supplementary Fig. S1). Others have also shown that myoblasts from MH mouse models differentiate and have functioning excitation-calcium release coupling by day 3.^26^

### XFe96 Seahorse assay

Experiments were performed using the manufacturer’s recommendations for the Seahorse XFe96 Substrate Oxidation Stress Test Kits (Agilent Technologies). Briefly, the XFe96 assay cartridge was hydrated overnight in distilled water followed by 1 h in XFe calibrant at 37°C in a non-CO_2_ incubator. Myotubes were washed and placed in warmed assay medium consisting of Agilent Seahorse XF DMEM media containing 10 mM glucose, 1 mM pyruvate, and 2 mM glutamine, pH 7.40 for 1 h at 37°C in a non-CO_2_ incubator, washed then transferred into the XFe96 Seahorse analyser. Drugs were added based on the manufacturer’s recommended protocol using 2.5 μM oligomycin, 1.5 μM carbonyl cyanide-p-trifluoromethoxyphenylhydrazone (FCCP), and 0.5 μM rotenone/antimycin A, and either 4 μM etomoxir, 2 μM UK5099, 3 μM N,N'-((thiobis(ethane-2,1-diyl))bis(1,3,4-thiadiazole-5,2-diyl))bis(2-phenylacetamide) (BPTES), or control assay media. All substrate inhibitors were added to the same plate to allow comparisons within and between genotypes. Standard substrate oxidation stress test assay parameters were used. Data analysis was performed using Seahorse analytics online software (https://seahorseanalytics.agilent.com), Microsoft Excel (V16.7, Microsoft, Redmond, WA, USA), and Prism 10.0 (Graphpad, Boston, MA, USA). Results represent four to six wells per genotype per condition that were repeated over six plates undertaken on different days, with a total of 28–32 wells.

The following formulae were used to calculate the variables reported in the results:

• Basal OCR=OCR pre-oligomycin−OCR after adding rotenone and antimycin A.
• Maximal OCR=Maximum OCR after adding 1.5 mM FCCP−OCR after adding rotenone and antimycin A.
• Normalised OCR (%)=Basal OCR/Maximal OCR×100%.
• Spare respiratory capacity normalised to FCCP=(OCR after FCCP−OCR pre-oligomycin)/Maximal OCR×100%.
• Proton leak=OCR after oligomycin−OCR after rotenone and antimycin A.
• Normalised proton leak=proton leak/Maximal OCR×100%.
• Normalised ATP linked respiration=(OCR pre-oligomycin−OCR after oligomycin)/Maximal OCR×100%.
• Normalised non-mitochondrial respiration=OCR after adding rotenone and antimycin A/OCR after FCCP×100%.

### Statistical analysis

Q-Q plots were used to assess whether data had a normal distribution for statistical comparisons, with nonparametric data assessed using Kruskal–Wallis test with Dunn’s multiple comparisons test. *P*<0.05 was considered statistically significant. Parametric data are reported as mean with 95% confidence interval (CI) (unless indicated), and nonparametric data as median with interquartile range (IQR) and range.

## Results

### The effect of *Ryr1* genotype on the oxygen consumption rate

**Fig 1:**
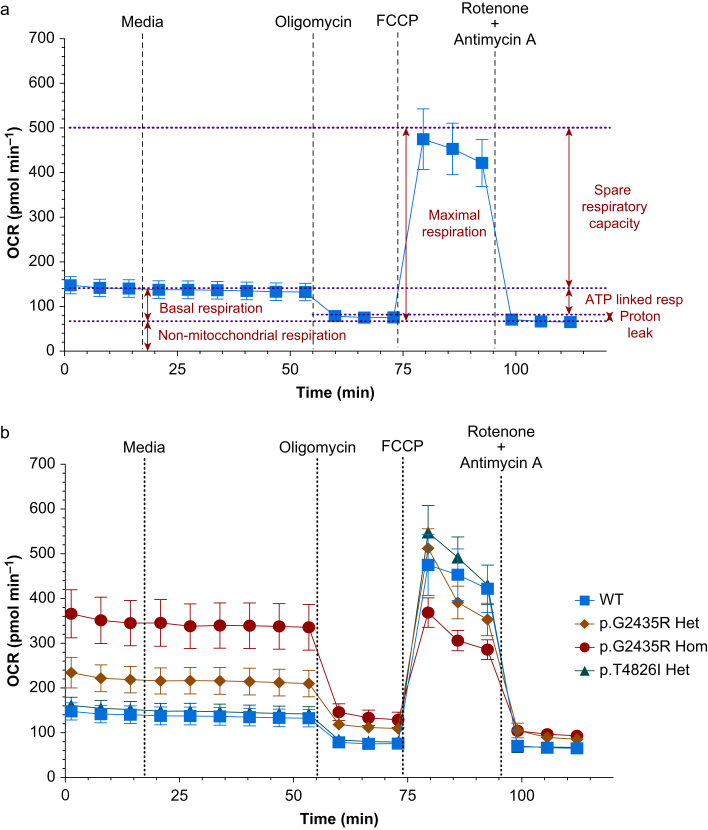
**Effects of *Ryr1* genotype on the basal and maximal oxygen consumption rates in MH myotubes.** (a) Oxygen consumption rate (OCR) from WT myotubes after addition of oligomycin (2.5 μM), FCCP (1.5 μM), and rotenone and antimycin A (0.5 μM). Mitochondrial bioenergetic parameters identified using the XFe96 Seahorse assay are indicated. (b) OCR observed in each genotype using the Seahorse assay. Data represent mean OCR (95% confidence interval), *n*=28–32 wells over six plates per genotype. FCCP, carbonyl cyanide-p-trifluoromethoxyphenylhydrazone; ATP, adenosine triphosphate; WT**,** wild-type.

**Fig 2:**
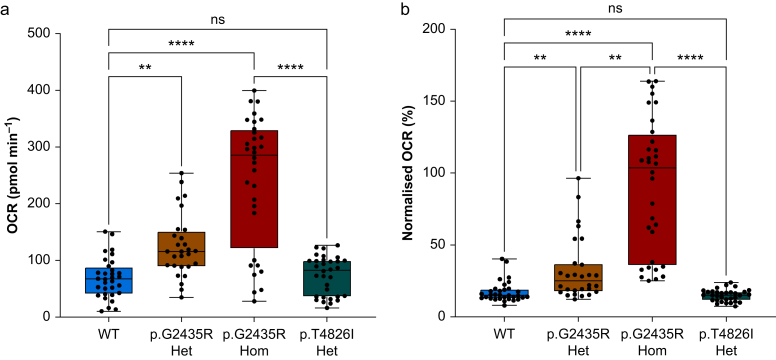
**p.G2435R *Ryr1* variants result in a gene-dose dependent increase in the normalised basal oxygen consumption.** (a) Box and whisker plots showing basal OCR for each genotype during the Seahorse assay. Data represent median OCR (interquartile range and range), *n*=28–32 wells per genotype. Genotype had a significant effect on the basal OCR, with p.G2435R Hom having the greatest basal OCR. ∗∗*P*<0.01, ∗∗∗∗*P*<0.0001, *n*=28–32. (b) Data from (a) normalised to maximal mitochondrial OCR achieved for each genotype after addition of 1.5 μM FCCP. There was a gene-dose-dependent effect on the normalised OCR with p.G2435R *vs* WT, but no difference between WT and p.T4826I Het. ∗∗*P*<0.01, ∗∗∗∗*P*<0.0001, *n*=28–32. OCR, oxygen consumption rate; WT, wild-type.

The OCR (pmol min^−1^) was determined for each genotype under different conditions of mitochondrial activity (Fig. 1a and b). Genotype had a significant effect on the basal OCR, with p.G2435R Hom exhibiting the highest OCR with a median of 286 (IQR, 121–330) pmol min^−1^, compared with p.G2435R Het at 116 (89–151) pmol min^−1^, p.T4826I Het at 83 (36–100) pmol min^−1^, and WT at 67 (41–88) pmol min^−1^ (*P*<0.0001, Fig. 2a). To account for the well-to-well variation in OCR because of myotube number, basal OCR was normalised to the maximal OCR achieved in each well per genotype. This also revealed a genotype-dependent change in normalised OCR (*P*<0.0001, Fig. 2b). After correction for multiple comparisons, p.G2435R Hom had a significantly (*P*<0.001) higher basal OCR with a median of 104% (35–127%) of maximal uncoupled mitochondrial respiration (Fig 1, Fig 2b); this was greater than that seen in p.G2435R Het 25% (18–37%), p.T4826I Het 15% (12–17%), and WT 15% (13–19%). However, the maximal OCR achieved in p.G2435R Hom was lower than that seen in the other three genotypes (Fig. 1B, Supplementary Fig. S2a).

**Fig 3:**
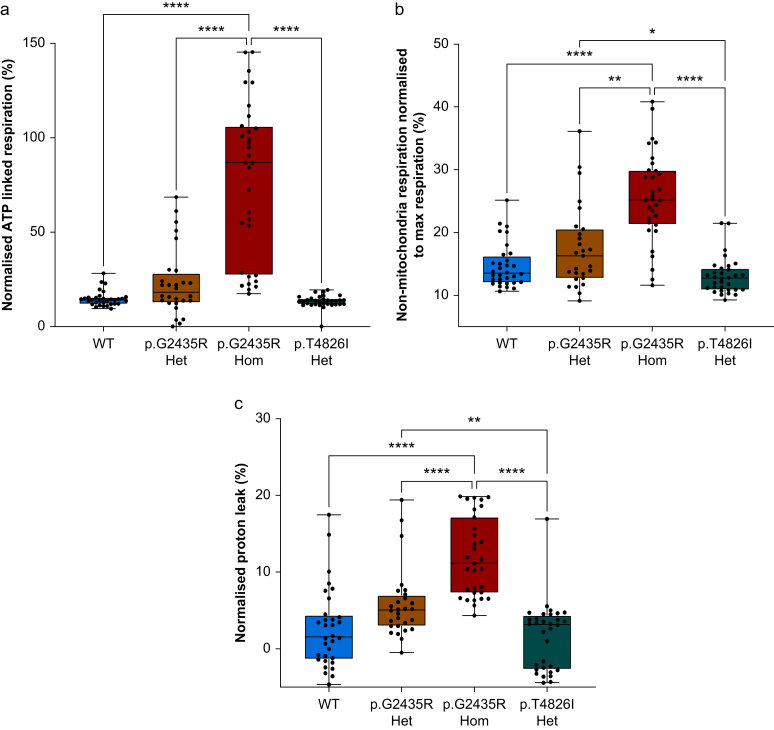
**p.G2435R Hommyotubes show distinct mitochondrial bioenergetics relative to control and other MH *Ryr1* variants.** (a) Graph displaying median and range of normalised ATP-linked respiration in each genotype. (b) Median and range of normalised non-mitochondrial respiration in the four genotypes. (c) Median and range of normalised proton leak per genotype. ∗*P*<0.05, ∗∗*P*<0.01, ∗∗∗∗*P*<0.0001, Kruskal–Wallis with Dunn’s multiple comparison test, *n*=27–32 per genotype. ATP, adenosine triphosphate; WT, wild-type.

To identify the proportion of basal OCR linked to ATP production, we calculated the difference in OCR before and after oligomycin and normalised to maximal uncoupled OCR (Fig. 3a). These data revealed that in p.G2435R Hom myotubes, a greater proportion of the basal respiration was dedicated to ATP production to meet their energetic demands (*P*<0.0001, Fig. 3a), with a median of 87% (27–106%), compared with 18% (13–28%) in p.G2435R Het, 13% (12–15%) in p.T4826I Het, and 14% (12–15%) in WT. In addition to increased basal mitochondrial oxygen consumption, p.G2435R Hom had greater normalised non-mitochondrial OCR with a median of 25% (21–30%) of the total maximum OCR (Fig. 3b), compared with 16% (13–21%) in p.G2435R Het, 13% (11–14%) in p.T4826I Het, and 14% (12–16%) in WT (*P*<0.01). The greater basal OCR together with reduced uncoupled OCR meant that p.G2435R Hom had reduced normalised spare respiratory capacity compared with the other three genotypes (Supplementary Fig. S2b).

p.G2435R Hom myotubes also had greater normalised proton leak of 11% (7–17%) compared with 5% (3–7%) in p.G2435R Het, 3% (−3% to 4%) in p.T4826I Het, and 2% (−1% to 4%) in WT (*P*<0.05, Fig. 3c). The proton leak represents the basal respiration that is not used directly in ATP production.

### The effect of substrate on oxygen consumption rate

**Fig 4:**
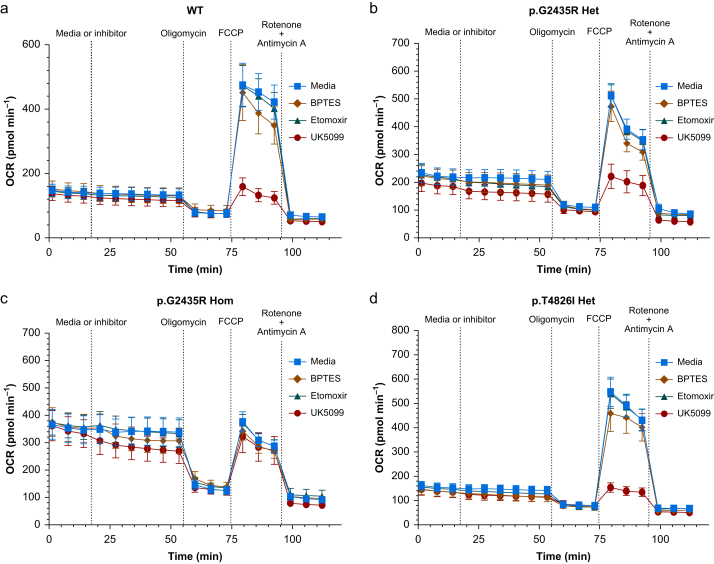
**Changes in oxygen consumption in each genotype following the addition of inhibitors**. These included inhibitors of the mitochondrial transport of glutamine (3 μM BPTES), long chain fatty acids (4 μM etomoxir), and glucose/pyruvate (2 μM UK5099). Data represent mean OCR (95% confidence interval), *n*=28–32 wells per genotype. Data are for myotubes from WT (a), p.G2435R Het (b), p.G2435R Hom (c), and p.T4826I Het (d). BPTES, N,N'-((thiobis(ethane-2,1-diyl))bis(1,3,4-thiadiazole-5,2-diyl))bis(2-phenylacetamide); FCCP, carbonyl cyanide-p-trifluoromethoxyphenylhydrazone; OCR, oxygen consumption rate; WT, wild-type.

**Fig 5:**
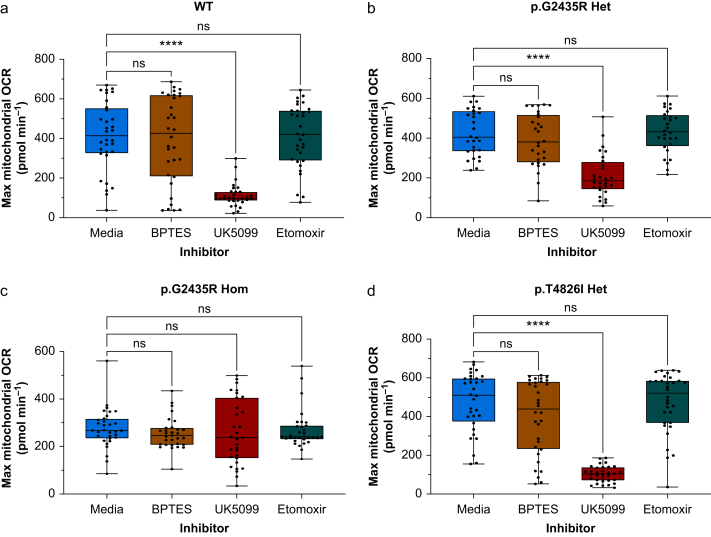
**Effects of substrate inhibitors on maximal oxygen consumption**. Data are shown for treatment with 1.5 μM FCCP in (a) WT, (b) p.G2435R Het, (c) p.G2435R Hom, and (d) p.T4826I Het myotubes. Mitochondrial transport of substrates was inhibited using 3 μM BPTES for glutamine, 2 μM UK5099 for glucose/pyruvate, and 4 μM etomoxir for long chain fatty acids. Data represent median and range of OCR, *n*=28–32 wells per genotype. ∗∗∗∗*P*<0.0001, Kruskal–Wallis with Dunn’s multiple comparison test. BPTES, N,N'-((thiobis(ethane-2,1-diyl))bis(1,3,4-thiadiazole-5,2-diyl))bis(2-phenylacetamide); OCR, oxygen consumption rate; WT, wild-type.

The OCR was determined for each genotype in the presence of control media, or inhibitors of mitochondrial substrates using 3 μM BPTES for glutamine, 2 μM UK5099 for glucose/pyruvate, and 4 μM etomoxir for long chain fatty acids (Fig 4, Fig 5). The basal OCR in each genotype was not affected by the presence of any of the three substrate inhibitors relative to control (Supplementary Fig. S3). At maximal OCR (following addition of 1.5 μM FCCP), there was no significant change in the OCR of each genotype relative to the control media after addition BPTES or etomoxir (Fig 4, Fig 5, *P*<0.05).

However, 2 μM UK5099 induced a decrease in the OCR relative to control media in WT from a median of 414 (IQR 326–553) to 98 (86–130) pmol min^−1^, p.G2435R Het of 405 (334–610) to 186 (143–279) pmol min^−1^, and p.T4826I Het of 511 (375–596) to 104 (70–187) pmol min^−1^ (*P*<0.0001), but not in p.G2435R Hom: 267 (235–316) to 238 (151–405) pmol min^−1^ (Fig. 5, *P*>0.05). Thus, WT, p.G2435R Het, and p.T4826I Het myotubes appear to be solely dependent on the glucose/pyruvate pathway to facilitate the increase in their mitochondrial capacity as they become uncoupled. In contrast, none of the substrate inhibitors including UK5099 had any significant effect on OCR in p.G2435R Hom when the cells were uncoupled (Fig 4, Fig 5c).

## Discussion

We assessed mitochondrial bioenergetics in myotubes cultured from three different mouse models of MH (*Ryr1* p.G2435R Het or Hom and p.T4826I Het). We had previously published data on the same MH mouse models using permeabilised skeletal muscle fibres; however, the methodology adopted was limited in throughput, was labour-intensive, and required high mice numbers.^20^ Utilising the Seahorse analyser, we produced data consistent with previous observations with greater sensitivity to genotype-dependent variation, validating the use of *Ryr1* p.G2435R cultured myotubes instead of muscle fibres for the study of mitochondrial bioenergetics.

Moreover, we hypothesised that the degree of change in mitochondrial bioenergetics is influenced by intracellular [Ca^2+^] in the myoplasm. The p.G2435R mouse model is known to have higher measured resting myoplasmic [Ca^2+^], with greater basal mitochondrial activity than that observed in WT.^2^^,^^20^ Basal mitochondrial activity was influenced in a gene-dose-dependent manner but was not directly related to myoplasmic Ca^2+^ given that p.T4826I Het mice have an even higher myoplasmic [Ca^2+^],^14^ yet their basal normalised OCR is not significantly different to WT.^19^

Our data show that mitochondria in p.G2435R Hom mice have lower maximal respiratory capacity compared with other genotypes, and that their basal ventilatory frequency is higher, working at maximal capacity. These observations are consistent with data from Chang and colleagues^20^ showing a higher baseline mitochondrial respiration rate in the muscle fibres of p.G2435R Hom mice after normalising to electron transport chain capacity. We also measured the proportion of basal respiration rate linked to ATP production and non-mitochondrial respiration, both of which were higher in the myotubes from p.G2435R Hom mice. Together, this evidence shows that the homozygous *Ryr1* p.G2435R variant greatly increases overall basal metabolic activity in myotubes in comparison with both WT mice and heterozygous counterparts.

Although our p.G2435R mouse data align with previous work,^20^ the results from the p.T4826I Het mice do not support all previous findings. Barrientos and colleagues,^19^ also using the Seahorse analyser, found lower respiratory capacity and basal respiration rates in the myotubes of p.T4826I Het mice, whereas our results were indistinguishable from WT mice. One factor that might explain the variability between studies in how chronic elevated cytosolic [Ca^2+^] leads to perturbations in mitochondrial bioenergetics is the age of animals used. We and Chang and colleagues^20^ used tissue from <3-month-old mice (myotubes or skeletal muscle fibres, respectively), whereas the skeletal muscle fibres used by Barrientos and colleagues^19^ were from 3–6-month-old mice, and fibres from 12–20-month-old mice by Yuen and colleagues.^14^ The latter reported late-onset mitochondrial swelling and abnormal distribution at or above 12 months of age. Such changes were not evident in fibres from younger mice and were dependent on skeletal muscle type; however, studies specifically comparing myotubes from older and younger mice were not performed.^14^ Durham and colleagues^16^ also found major changes in mitochondria architecture in skeletal muscle fibres from 12-month-old *Ryr1* p.Y522S mice (a model also shown to have both raised myoplasmic [Ca^2+^] and basal oxygen consumption), but less so in fibres from younger 2-month-old mice. Therefore, age could prove to be an important cofactor that needs to be considered in future studies investigating the effects of pathogenic RyR1 variants linked to MH on mitochondrial bioenergetics. It is important to note that myotubes are less developed skeletal muscle cells relative to skeletal muscle fibres, with the former having more active bioenergetics.^27^ Although ageing has been shown to cause different effects on the mitochondrial bioenergetics of myotubes and fibres, both show consistent changes during the ageing process, with reductions in oxidative capacity a common finding.^28^, ^29^, ^30^ The age-related similarities between fibres and myotubes in mouse models appear to be attributable to the common origin of the mitochondria in both cell types, age-related reductions in regulatory factors, and the age-related environmental stressors that both are exposed to. These lead to impaired mitochondrial biogenesis, increased oxidative stress, greater mitochondrial DNA damage, and disrupted mitochondrial dynamics.^27^^,^^28^^,^^30^, ^31^, ^32^

Other possible explanations include methodological differences used in the studies; our protocol used a 10-fold higher cell density as preliminary experiments revealed this produced a greater signal-to-noise ratio. We further limited inter-well cell variability by reducing the myoblast proliferation time by 50% before initiating differentiation. Reagent concentrations also differed between studies, as Barrientos and colleagues^19^ used a five-fold higher concentration of oligomycin, 33% less FCCP, and 80% less rotenone, whereas antimycin A was not used. A higher concentration of FCCP is more likely to uncouple all cells sufficiently to reach the maximal OCR for that genotype; therefore, p.T4826I Het reached their true maximal OCR and were not different from WT. Furthermore, use of only rotenone without antimycin A might incompletely inhibit the electron transport chain, particularly at 0.1 μM rotenone.^24^

In comparison with other mouse genotypes, p.G2435R Hom myotubes exhibited increased proton leak. Chang and colleagues^20^ found p.G2435R Hom had greater proton leak compared with p.T4826I Het mice, but failed to detect differences against WT and p.G2435R Het. We were able to achieve greater resolution between genotypes in the Seahorse analyser. Proton leak refers to the transfer of protons across the inner mitochondrial membrane which dissipates energy into heat as opposed to ATP synthesis^33^ as a measure of mitochondrial coupling efficiency, and is associated with regulation of ROS production.^34^ Mitochondrial ROS is produced within the electron transport chain which links ROS turnover rates to ATP production.^35^ The p.G2435R mouse model has been found to exhibit a gene-dose-dependent increase in basal ROS levels, which aligns with the increased ATP-linked respiration observed in our study.^20^ Increases in proton leak regulate mitochondrial function in the presence of high ROS levels by uncoupling the system and reducing the efficiency of electron transport through the chain.^33^^,^^36^

The Seahorse assay allowed us to develop a bioenergetic profile of MH cells, with p.G2435R Hom displaying a greater degree of dysfunction with a lower Bioenergetic Health Index (BHI), a novel dynamic measure of the response of the body to stress.^37^ Healthy subjects have a higher BHI with greater reserve capacity, high ATP-linked respiration, and low proton leak. Chronic cell stress leads to damaged inefficient mitochondria and deterioration in these measures. The low BHI seen in p.G2435R Hom cells is likely a result of increased cellular stress caused by abnormal RyR1. Higher basal metabolic activity is likely driven by higher myoplasmic [Ca^2+^] causing an increase in sarcoendoplasmic reticulum calcium ATPase (SERCA) pump activity to transport Ca^2+^ from the cytosol back to the SR. Damage from ROS and RNS to mitochondria and associated impairment of oxidative phosphorylation lead to increased inefficiency that requires increased mitochondrial work resulting in greater oxygen consumption during ATP production. The inefficiencies also render the cells less able to adapt to acute or chronic cellular stress and might underlie the phenotypic observations of reduced exercise tolerance, greater muscle fatiguability, and increased exertional heat illness seen with MH-associated *RYR1* variants.^38^^,^^39^

Reduced BHI together with changes in substrate metabolism could also explain why patients susceptible to MH have a greater incidence of impaired glucose metabolism with greater incidence of hyperglycaemia and glycosylated haemoglobin.^40^^,^^41^ If age is found to be an important factor, then one could infer that the mitochondria-driven muscle weakness phenotype in MH patients might be later-onset and less apparent in young cases. Further high-throughput cell-based studies using both animal models with RyR1 agonists and antagonists, and human MH cells in the Seahorse analyser, would help elucidate correlations between mitochondrial bioenergetics and human phenotypes.

The substrate inhibitor assays confirmed pyruvate/glucose to be the main mitochondrial substrate in WT, p.G2435R Het, and p.T4826I Het myotubes, accounting for 55–80% of basal respiration rates. However, this was not observed in p.G2435R Hom myotubes where the OCR was unaffected after inhibition of the mitochondrial pyruvate carrier. A metabolomic study on MH-susceptible patients suggested a shift in substrate usage from carbohydrates to lipids,^8^ which led us to speculate whether p.G2435R Hom myotubes behave in a similar manner. This was not observed in any of the MH mouse genotypes given etomoxir, which did not impact basal or maximal respiration rates associated with fatty acid utilisation. Likewise, the glutamine transport inhibitor BPTES did not have an effect in any of the genotypes studied. Together, these data indicate that at <3 months of age, elevated myoplasmic [Ca^2+^] appears to have differential consequences on mitochondrial bioenergetics in different MH mouse models, suggesting the existence of different compensatory mechanisms for the cellular stress. Future studies involving myotubes derived from mice harbouring other pathogenic RyR1 variants such as p.R163C Het/Hom and p.T4826I Hom and from young and aged mice would help elucidate the role of these variants in a gene-dose- and age-dependent manner on mitochondrial bioenergetics.

Although the OCR in p.G2435R Hom myotubes was insensitive to the glucose/pyruvate pathway upon uncoupling, on closer examination, there seems to be another factor that leads to impairment in the ability of this genotype to increase OCR when oxidative phosphorylation is uncoupled, therefore resulting in lower spare respiratory capacity. Spare respiratory capacity is a marker of mitochondria fitness, and reflects the mitochondrial capacity to meet extra energy requirements above the basal level during acute stress or heavy workload, thereby avoiding an ATP crisis.^37^^,^^42^^,^^43^ Alterations in mitochondrial structure are seen in unhealthy mitochondria which also have reduced spare respiratory capacity. Consistent with this and our findings of a reduced spare respiratory capacity in p.G2435R Hom, this genotype has larger and more globular mitochondrial ultrastructure compared with normal in p.G2435R Het and WT.^21^ This was associated with elevated mitochondrial Ca^2+^ content. However, the mechanisms linking these changes in mitochondrial ultrastructure and bioenergetics in MH cells are yet to be elucidated.

In conclusion, our data confirm a lower maximal respiratory capacity in combination with greater basal metabolic rates in the myotubes of p.G2435R Hom mice. We speculate that the increased proton leakage observed in p.G2435R Hom mice is acting as a compensatory mechanism to protect cells from high ROS concentrations. Finally, the use of cultured myotubes in the Seahorse analyser improves data resolution and generates findings consistent with muscle fibre respirometry,^20^ which validates this approach as an alternative to help reduce the burden on animal models in future studies.

## Authors' contributions

Conception and design of the study, data analysis and interpretation, review of drafts of the manuscript and approval of the final version: all authors

Conduct of experiments and data collection: VK

Drafting of manuscript: VK, LC

## Funding

*British Journal of Anaesthesia*/Royal College of Anaesthetists (grant WKR0-2021-0003 to PMH and MAS); VK is grateful for an institutionally funded National Institute for Health and Care Research academic clinical lectureship in anaesthesia.

## Declarations of interest

PMH is former editor-in-chief of *BJA Open* and an editorial board member of the *British Journal of Anaesthesia*. The other authors declare that they have no conflicts of interest.
