# Supplementary material for: Mitochondrial bioenergetics and intracellular calcium concentration in primary myotubes from mouse models of malignant hyperthermia
Source: Br J Anaesth. 2025 Aug 19;136(1):333–42. doi: 10.1016/j.bja.2025.05.060 (PMC12851885; doi:10.1016/j.bja.2025.05.060)
Supplement: Multimedia component 1 [file mmc1.docx]

**Supplementary Figure 1:** Representative images showing that all four genotypes produce multinucleated cells which are visible on brightfield microscopy. Immunofluorescence with 1:200 of Rabbit anti-mouse myosin heavy chain 3 (Ab124205, Abcam, UK) and 1:1000 goat anti-rabbit immunoglobulin G heavy and light chain antibody with Alexa-Fluor 488 (Ab150077, Abcam, UK) confirmed the expression of myosin heavy chain (green) in the myotubes with multiple nuclei evident in each myotube following staining with 300 nM 4′,6-diamidino-2-phenylindole (DAPI, blue).
