# Supplementary material for: Mitochondrial bioenergetics and intracellular calcium concentration in primary myotubes from mouse models of malignant hyperthermia
Source: Br J Anaesth. 2025 Aug 19;136(1):333–42. doi: 10.1016/j.bja.2025.05.060 (PMC12851885; doi:10.1016/j.bja.2025.05.060)
Supplement: Multimedia component 2 [file mmc2.docx]

**A**

**B**

**Supplementary Figure 2: (A)** The maximal median respiration and range achieved in each genotype following the addition of 1.5 µM FCCP. **(B)** Normalised spare respiratory capacity in the four genotypes. ****P<0.0001, ***P<0.0005 and **P<0.01, n=28-32**.**
