# Supplementary material for: Mitochondrial bioenergetics and intracellular calcium concentration in primary myotubes from mouse models of malignant hyperthermia
Source: Br J Anaesth. 2025 Aug 19;136(1):333–42. doi: 10.1016/j.bja.2025.05.060 (PMC12851885; doi:10.1016/j.bja.2025.05.060)
Supplement: Multimedia component 3 [file mmc3.docx]

**Supplementary Figure 3:** The effect of the inhibitors of the glucose/pyruvate (UK5099), long-chain fatty acid (etomoxir) and glutamine (BPTES) pathway on the basal OCR in WT **(A)**, p.G2435R Het **(B)**, p.G2435R Hom **(C)**, and p.T4826I Het **(D)**. P>0.05, Kruskal-Wallis with Dunn’s multiple comparison test, n=28-32 wells per genotype.
